# Cognition and wellbeing in middle-aged early treated people with phenylketonuria: Preliminary results and methodological lessons

**DOI:** 10.1016/j.ymgmr.2024.101160

**Published:** 2024-11-21

**Authors:** Lucie Thomas, Lynne Aitkenhead, Karolina M. Stepien, Alison Woodall, Anita Macdonald, Cristina Romani

**Affiliations:** a Aston University, UK; b University College London Hospitals, UK; c Salford Royal Organization, Northern Care Alliance NHS Foundation Trust, UK; d Birmingham Women's and Children's NHS Foundation Trust, UK

**Keywords:** PKU, Aging, Cognition, Wellbeing, Quality of life

## Abstract

The first cohort of early-treated adults with phenylketonuria (PKU) is reaching middle-age and moving towards old age. We do not know if and how the effects of an aging brain may interact with the effect of PKU. This study compared wellbeing and cognition in 19 middle-aged adults with PKU (age 40+ mean = 45.8) and in a younger adult PKU group (age 18–36 mean = 26.7). The middle-aged PKU group demonstrated more anxiety and depression, and more negative effects of the COVID-19 pandemic, compared to age-matched controls. They also demonstrated a steep deterioration of quality of life compared to younger adults with PKU. These last results confounded age with the effects of the pandemic, since only the older participants were tested during the COVID-19 pandemic, but taken together, results consistently point to AwPKU being less resilient to age and other life stressors affecting wellbeing. Regarding cognition, the older PKU group demonstrated significantly worse performance than the younger group, and within the middle-age groups, the effect of age was stronger in the PKU group than in the control, even though this was not statistically significant. In contrast, size of impairment relative to an age-matched control group was numerically smaller in older, middle-age PKU group. We discuss possible methodological confounders related to this last result. Our study points to the challenges of using cross-sectional results to track performance across the lifespan and to the need to acquire more corroborating evidence before concluding there is no accelerating brain aging in PKU.

## Highlights

• In early treated middle-aged adults with PKU, wellbeing was worse than in age-matched controls;
• Cognitive performance was worse than in younger adults with PKU;
• The pattern of cognitive deterioration was similar to healthy controls;
• The effect of age was numerically stronger in the middle-aged PKU group than in controls;
• Performance relative to controls did not worsen with age, but this may be due younger and older controls not being well matched;
• Studies with better matching of clinical and control groups, with more representative PKU groups and with older cohorts are needed.

## 1 Introduction

Phenylketonuria (PKU) is an inherited metabolic disease where a defect in coding for the enzyme, phenylalanine hydroxylase (PAH) results in an inability, or severely reduced ability, to metabolize the amino acid phenylalanine (Phe) into tyrosine. The accumulation of Phe in the blood and in the brain results in severe intellectual disability in untreated people with PKU. The introduction of a severely Phe-restricted diet in newborn children with PKU since the early sixties has allowed people with PKU to lead fulfilling lives, but with clear limitations (https://phescreening.blog.gov.uk/2020/01/15/blood-spot-screening-50) and current treatment options and outcomes are suboptimal [[1], [2], [3], [4]]. Pharmaceutical treatments, although in continuous development, cannot be accessed and/or are not an effective option for everyone, while dietary treatment is costly, burdensome, and difficult to adapt in social circumstances. Thus, because it is increasingly difficult to manage with increasing age, the PKU diet is relaxed or abandoned by a large proportion of people with PKU after adolescence [5,6] despite recommendation for continuous treatment [7].

The first cohort of early treated adults with PKU (AwPKU) has now reached middle-age and we need to review outcomes to evaluate the possible impact of protracted elevated Phe levels. We need to know how middle-aged AwPKU are performing in terms of cognitive processing and wellbeing to reassure us that no accelerated deterioration is detectable at this stage, and to have a baseline against which to compare outcomes later, when old age is reached, and brain-health and general health declines. With these aims, our study assessed outcomes in a group of middle-aged AwPKU and compared results with an aged-matched control group and with a group of younger AwPKU. Moreover, since our study was carried out during the COVID-19 pandemic, we also aimed to assess the impact of the pandemic on people with PKU.

There is some consensus that developing brains are more susceptible to the negative effects of Phe than adult brains. This is reflected in the European guidelines which recommend keeping Phe <360 mmol/L before 12 years and < 600 μmol/L, above 12 years [7,8] (American guidelines recommend 120–360 μmol/L throughout life [9,10]). One may wonder if enhanced sensitivity to Phe would also occur in aging brains. Several results point to possible risks in running Phe levels systematically above 600 μmol/L, even after adolescence, and suggest caution, although counterarguments can also be presented limiting the force of these results. We review this evidence.

### 1.1 Cognition

Correlations between current Phe levels and cognitive performance have been demonstrated not only in children but also in AwPKU [[11], [12], [13], [14]]. The force of these results, however, is limited by correlations being present only for some cognitive tasks (tapping a combination of abilities including sustained attention, flexibility, short-term memory, and speed of processing) and, more importantly, by the fact that these results do not consider that adult Phe and childhood Phe may be strongly correlated. Thus, those people with high adult levels may be the same who had high levels when children, so that child levels, rather than adult levels, may be responsible for a depressed cognitive performance (but see for example Aitkenhead et al., 2021 for correlations between current Phe level and performance in the symbol-digit task, even after partialling out Phe levels at previous ages [11]).

An increasing number of studies have reported cases of AwPKU who have manifested a sudden and catastrophic deterioration of cognitive performance and/or the onset of neurological symptoms in spite of having stable cognition and elevated Phe levels for many years (some were people with undetected, and therefore untreated, PKU; others were early-treated people with PKU who had abandoned the diet for many years [15]). These results, together with those above, suggest that elevated Phe levels across a long period of time could have cumulative negative effects. On the other hand, one may argue that these cases are exceptional, and, in the great majority of people, cognitive outcomes remain stable regardless of adult Phe levels.

Probably the strongest evidence that Phe affects cognition even in adults comes from intervention studies where cognitive performance is compared within participants when Phe levels are different. These studies have generally shown better performance with lower Phe levels [16]. Two more recent studies are important in pointing to possible parameters of change. Trepp et al. (2024) [17] have carried out a double-blind study with 26 early treated AwPKU. They were administered either a placebo or Phe-loading capsules (which would raise Phe to the levels of an unrestricted diet; around 1400 μmol/L) for two alternating 4-week periods. Results showed that only sustained attention (measured as SD in an n-back task) significantly worsened in the Phe period, while other measures (accuracy and RT in the n-back task, manual dexterity, and measures of mood) did not change. In contrast to these results, Manti et al. (2023) [18] have shown dramatic improvements in a small group of women planning a pregnancy (*N* = 7) when Phe was lowered close to physiological levels. Performance was measured at baseline (average Phe = 1311 μmol/L; SD = 278) and then when Phe was lowered and maintained <360 for three months (average Phe =166; SD = 64) and, again, after 3 further months (average Phe =162; SD = 51). Improvements occurred across a wide range of functions and tasks and were very significant after both the periods when Phe was lower, corresponding to on average 0.98 and 1.68 standard deviation improvements compared to baseline performance. These results suggest a strong effect of Phe, even in adulthood, when near physiological levels are contrasted with levels associated with an unrestricted diet and when low Phe is maintained for a sufficiently long period of time.

Regarding neurophysiological measures and their relation to cognition several studies have evidenced increased white matter deterioration with increased age [[19], [20], [21], [22], [23], [24], [25]] and a relation with Phe levels [20,[26], [27], [28]], but no parallel cognitive deterioration [20,[22], [23], [24], [25]]. These studies, however, have only considered young participants (differences between children and adolescents and/or between adolescents and young adults). We do not know whether neurophysiological abnormalities will remain asymptomatic or whether they will be associated with cognitive impairments when combined with the effects of normal aging. (and for results showing a positive association between white matter damage and executive difficulties see Antenor-Dorsey et al., 2013; Muri et al., 2023. [29,30]).

Finally, only the studies by Weglage et al. (2013) [31] and Feldmann et al. (2019) [32], that we know of, have compared cognitive performance across young and more mature adults. They followed longitudinally *n* = 35 AwPKU from an average age of 32 years to an average age of 42 years, testing them at two 5-year intervals with IQ, the trial-making-test (TMT)) measuring flexibility and visuo-motor control, and the d-2 test measuring sustained attention. Performance remained stable.

Taken together several lines of evidence suggest that Phe levels continue to affect cognition and brain health throughout adulthood and point to the importance of carefully considering possible interacting effects of aging. The results of the studies by Weglage et al. (2013) [31] and Feldmann et al. (2019) [32] are encouraging in showing no increased cognitive difficulties, but more evidence is needed.

### 1.2 Wellbeing

Mental wellbeing can also be disproportionally impacted by aging in AwPKU and should be considered. Under the umbrella of wellbeing, we will consider both quality of life and mental health (depression, anxiety, mood). In AwPKU, difficulties in these areas are less strong and systematic than cognitive difficulties [33]. Some studies have reported reduced quality of life and/or correlation with metabolic control [34,35], but others have found no increased difficulties and no correlation [11,33,36]. Quality of life investigated with an ad-hoc, PKU specific questionnaire has also revealed little difficulty [34,[37], [38], [39]] although increased difficulties have been noted in association with higher Phe and lower cognition [38]. Similarly, results considering mental health have been mixed. Some studies have reported reduced mental health [[40], [41], [42]], associations with Phe (either childhood Phe [43,44], or life-time Phe levels [45]), and increased difficulties when Phe was pharmacologically increased [46]. Other studies, however, have reported no increased anxiety or depression in AwPKU and no correlations with metabolic control [11,35,36]. Even if young AwPKU generally demonstrate good wellbeing, however, it is clear that managing the diet is difficult [11] and that PKU is linked to comorbidities which may increase with age [47]. These difficulties may interact with other life-stressors, such as living during a pandemic, to provoke a stronger deterioration in mental wellbeing with age than what is seen in healthy controls [40,48].

In our study, we considered cognition and wellbeing in 19 AwPKU aged 40+ (average age: 45.8; range = 40–56) in comparison with a 10-years younger cohort (average age: 26.7; range = 18–36). We tested cognition with a large range of tasks tapping different cognitive functions. We were particularly interested in differences in speed of processing since this is notoriously affected by age [[49], [50], [51], [52]]. Therefore, we administered several tasks where performance was assessed in terms of both RTs and accuracy. We assessed wellbeing using the SF-36 quality of life questionnaire [53] as well as the PKU Quality of Life Questionnaire [39]. We assessed depression and anxiety using the Beck Depression Inventory and the Beck Anxiety Inventory BAI [54];. Finally, we used an ad-hoc questionnaire to compare the impact of the COVID-19 pandemic on our middle-aged cohort of AwPKU and matched controls. We will explore associations of cognitive and wellbeing scores with blood-Phe concentrations. We want to stress from the beginning, however, that our comparisons are only preliminary. Cross-sectional, between-groups comparisons are not as reliable or powerful as longitudinal comparisons. Moreover, we will be comparing younger groups tested face-to-face with older groups who, because of the pandemic, were tested online. Studies have generally reported good consistency between modalities of testing [55], but it is possible that online recruitment biases may have affected the quality of performance of our control participants. Limitations will be addressed in the general discussion.

## 2 Method

### 2.1 Participants

We asked all potential participants (clinical and control) to self-exclude if they suffered from conditions affecting cognition and mental health such as a developmental, neurodegenerative or psychiatric disorders.

Nineteen early-treated AwPKU, age range 40 to 56 years, with classical PKU were recruited from the Charles Dent Metabolic Unit at University College Hospitals London (UCLH; *n* = 13) and the Mark Holland Metabolic Unit at Salford Royal Organization, Northern Care Alliance (NCA; *n* = 6). All participants were diagnosed through new-born screening conducted 5–7 days after birth and reported to have continuously followed a low-Phe diet from diagnosis until adolescence. Participants from both clinics were approached by dieticians or clinicians during their scheduled clinic visits (either in person or via telephone). Additional recruitment was conducted by Salford Royal through sending information to eligible patients via post. Interested participants then contacted the research team at Aston University for more information, and to provide consent to participate. Data on adulthood Phe levels was gathered from the UCLH and NCA patient databases. To measure current blood Phe, participants were asked to send a dried blood spot test card into the hospital on the same day that they completed their first assessment. In one instance where the blood spot test was not suitable for analysis, we took their most recent blood spot result, which had been completed within 2 weeks of taking part in the study.

At time of testing, six participants were on an unrestricted diet and 13 were following a restricted diet. All early-treated PKU individuals attending the clinic were invited to participate, as well as several individuals who were still contactable. Forty-one individuals responded to the invitation. However, 23 did not go on to take part due to a lack of time (*N* = 2), a lack of access to an appropriate device (*N* = 1), ill health (N = 1), changing their mind (*N* = 4), or not responding to further communications (*N* = 15). Twenty participants were consented and tested. One participant was later excluded as they did not receive treatment until 3 months after birth.

Healthy adults, matched to PKU participants for age, gender, and education were recruited through the Aston University volunteering website, and through adverts posted on social media and in public spaces (e.g., village halls, Aston University campus; *N* = 39). However, data from some participants was excluded due to concerns about the participants' truthfulness in answering background questions and their commitment to provide quality data (these participants formed a group who contacted researchers at about the same time) and others only completed one testing session. This left results from 27 control participants.

To compare results across ages, we used data from the young early-treated adults with classical PKU we had assessed in previous studies [12,13,56]. The original cohorts included 37 AwPKU and 30 controls. However, to comply with the age restriction of the present study where we wanted at least a 10-year age gaps between younger and older cohorts, we excluded three controls and four people with PKU. That left us with 33 young AWPKU and 27 controls. Demographic information for all the groups is reported in Table 1 (see original studies for more details). None of our PKU participants were pharmacologically treated for PKU (e.g., received Sapropterin or Pegvaliase).

**Table 1:** Demographic information for the young and older PKU and control groups.

		Middle-aged		Younger	
		AwPKU	Controls	AwPKU	Controls
	n	19	27	33	27
**Gender**	% female	42	70	64	73
**Age** **(in years)**	Mean	**45.8**	**46.5**	**26.7**	**26.2**
	*SD*	*4.4*	*4.3*	*6.9*	*6.4*
	Range	40–56	40–55	18–36	18–36
**Education** **(in years)**	Mean	**14.5**	**15.8**	**14.5**	**15**
	*SD*	*2.7*	*2.7*	*2*	*1.7*
**Metabolic control**					
**Phe** **Current** **(most recent value)**	Mean	**926**	–	**725**	–
	*SD*	*387*	–	*353.2*	–
	Range	1945–227	–	1465–65	–
	Median	912		492.5	
**Phe** **preceding decade**	Mean	**931**	–	**776**	–
	*SD*	*378*	–	325	–
	Range	1856–324	–	1310–290	–
	N values mean	18	–	58	–
	*SD*	15.4	–	71	–

All the research reported in this paper was approved by the NHS ethics committees (IRAS 255157). All participants gave voluntary informed consent to take part.

### 2.2 Materials

Remote assessments were designed to reflect the face-to-face assessments previously used by Palermo (2017) and Romani (2017) as closely as possible, although the online presentation required some differences [12,56]. Cognitive outcomes were assessed considering: **Verbal IQ** (using the vocabulary and similarities subtests of the WAIS), **Executive functions**: using the Wisconsin Card Sorting Test (*WCST*; 64-card version), Verbal fluency (both phonemic and semantic), the Illogical minus the logical condition in a sentence completion task (complete the sentence with an unrelated word); the incongruent minus the congruent condition of the Stroop task; **Memory and learning**: Digit span, Corsi span, Non-word repetition, Rey Auditory Verbal Learning Task, Picture-nonword paired associate task; **Visual attention**: Simple detection, Choice reaction time; Detection with distractors; Visual search (with feature and conjunction search conditions); **Sustained attention:** Rapid Visual Information Processing (RVP); **Language:** Picture naming (considering both RTs and accuracy), Word and non-word spelling, Phoneme deletion, Spoonerisms, Neutral and congruent condition of Stroop task (naming the colour of a string of XXs or, in the congruent condition, naming the colour of a word which spells the same colour). See Supplentary Materials A for details.

Quality of life was assessed using two questionnaires: the ***36-Item Short Form Health Survey*** SF-36 [53]; which assesses physical functioning, pain, limitations due to physical and emotional health, general emotional wellbeing, social functioning, energy, and general health perceptions; and the PKU ***quality of life questionnaire*** PKU-QoL [39]; which assesses PKU symptoms, management of PKU, intake of protein supplements and adherence to diet.

Mental health was measured with self-reporting questionnaires that assess depression (the *Beck **Depression Inventory**;* BDI-II [57]; and anxiety (the ***Beck Anxiety Inventory**;* BAI [54];.

The effect of the COVID-19 pandemic on health and emotional wellbeing was assessed with an ad hoc ***COVID-19 questionnaire**.* It included nine questions. Seven asked about stress, isolation, anxiety, exhaustion, sadness, anger, and concern about their health. They had to be answered on a 7-point Likert scale (from −3 to +3) with 0 indicating no change, positive scores (1−21) indicating more difficulty during the lock-down and negative scores indicating less difficulty (−1- (−21)). Two further questions probed management of PKU and access to supplements during the lockdown; scores 1–3 indicated that PKU management had become more difficult during the lockdown and negative scores −1 to −3 indicated that it had become easier.

All questionnaires were delivered through Qualtrics.

For more details on the cognitive assessment and on the questionnaire administered see Supplementary Materials.

### 2.3 Procedure

Testing was completed remotely over two to three video-call sessions (depending on individual requirements), each lasting a maximum of two hours with a break encouraged in the middle. Sessions were conducted either through Zoom or Microsoft Teams.

The first session involved eight computerized tasks. The researcher explained each task before sending the relevant link to experimental programmes through the chat function and was available to answer any queries during and after task completion. Online materials were created using the PsychoPy3 Experiment Builder and hosted online by Pavlovia. Online tasks included: Simple Reaction Time, Choice Reaction Time, Detection with Distractors, Visual Search, Wisconsin Card Sorting, Rapid Visual Processing (RVP), and Corsi Span tasks. All remaining tasks were carried out by interview via video link. Two further computerized tasks (Picture naming and Stroop) were hosted on Pavlovia.

A maximum of three weeks gap occurred between the testing sessions. Where possible, all participants were assessed on all measures. However, technical difficulties resulted in RT data being unavailable for 12 controls and seven AwPKU for the Stroop and picture naming tasks. Questionnaires probing quality of life were only administered to the PKU group and were compared to published norms.

### 2.4 Data handling and scoring

For each participant, from raw scores we calculated two kinds of z-scores. We calculated z-scores as deviation of the referent age-matched control population to estimate size of impairment, but we also calculated z-scores as deviations from the younger clinical/control population to estimate effect of aging. In both cases, z scores were computed as [individual score] – [average of comparison group]/[SD comparison group SD]).

Where necessary, z-scores were reversed to ensure that lower z-scores always reflected poorer performance. Individual participant reaction times (RTs) were cleaned to exclude errors and outliers (responses <100 ms or ± 3SD from participant mean). The result of one control participant in the detection with distractors task were removed from analysis, as their RT was >10 SD away from the mean.

Given the large number of tasks administered, to reduce noise and increase reliability, we calculated composite scores by averaging individual z-scores for related tasks. Composite scores for *speed of processing* were calculated by averaging z-scores across simple detection, choice RT, detection with distractors, feature search, conjunction search, RVP, Stroop, and picture naming tasks. A comparable composite *accuracy score* was calculated by averaging performance on the same tasks (except simple detection where performance was at ceiling).A composite *executive function score* was calculated by averaging mean z-scores for WCST error rate; sentence completion differences in errors and RTs between illogical and logical conditions; Stroop differences in errors and RTs between incongruent and congruent conditions; detection with distractors differences in error rates and RTs between the condition where the green bug was the target and the conditions where the ladybird was switched as the target. A composite *language score* averaged: Vocabulary WAIS subtest; Similarities WAIS subtest; Spelling words % errors; Spelling nonwords % errors; Phoneme deletion % errors; Spoonerism % errors. A composite *memory* score averaged:Digit span; Non-word repetition % errors; Corsi span; Picture non-word paired associates % errors; Pictured non-word paired associates delayed recall % errors; Rey A learning % errors; Rey A delayed recall % errors.

## 3 Results

### 3.1 Cognitive assessment

#### 3.1.1 Clinical comparison: PKU vs controls

Results for individual tasks for middle-aged AwPKU and control are reported in Supplementary Materials= B (Table 1S) which shows effect size of impairments from controls (Glass's Delta effect sizes). For most tasks differences do not reach significance. However, in tasks where performance is measured with RTs middle-aged AwPKU are systematically slower and there is overall a significant difference. There is also a borderline difference from controls for a task measuring sustained attention (RVP) and an overall significant difference when performance is considered across all tasks.

Table 2 compares indexes of impairment from age-matched control groups for middle-aged and younger AwPKU. Indexes of impairment for domains of interests are computed by averaging z-scores (from controls) across relevant tasks. Overall, the middle-aged PKU group showed a marginally significant impairment compared to controls. However, one can appreciate the contrast between clearly impaired speed of processing, but good performance with corresponding accuracy measures. Executive functions, language and memory do not demonstrate significant impairments. Note, however, that in our assessment, EFs have been measured mainly in terms of inhibitory control considering differences in performance between conditions which tax inhibitory control and baseline conditions. We have previously found no PKU impairment with these measures [3].

**Table 2:** Cognitive performance as difference from matched healthy control groups for middle-aged AwPKU and younger AwPKU.

INDEX of impairment	Middle-Aged AwPKU vs Middle-aged controls			Difference from controls	Young AwPKU vs young adults controls			Difference from controls	Difference impairment size younger vs older PKU
	n	Mean z-score	*SD*	p *t*-test	n	Mean z-score	*SD*	p *t*-test	p *t*-test
Speed of processing	19	**−0.47**	*0.9*	**.05**	33	**−0.88**	*1.2*	**<.001**	.19
Accuracy	19	**−0.16**	*0.5*	.45	33	**−0.28**	*0.7*	**09**	.51
Executive functions	19	**0.00**	*0.48*	.96	33	**−0.42**	*0.8*	**.03**	**.04**
Sustained attention	18	**−0.62**	*1.35*	**.08**	33	**−0.66**	*1.1*	**.01**	.90
Language	19	**0.07**	*0.8*	.69	33	**−0.47**	*1.0*	**.04**	.06
Memory	19	**−0.04**	*0.3*	.71	33	**−0.30**	*0.6*	**.02**	**.09**
Overall	19	**−0.22**	*0.35*	**.05**	33	**−0.40**	*0.5*	**<.001**	.16

Indexes of impairment are calculated by averaging individual participants' z-scores for different tasks. Z scores are calculated as the difference of an individual performance from the age-matched control group divided by the SD of the control group.

When size of impairment from control groups were compared for middle-aged and younger PKU groups, overall, there was no significant difference, but the middle-aged AwPKU showed a numerically smaller impairment (overall z-score from control group = −0.22; *p* = .05) than the younger PKU group (overall z-score = −0.40). In fact, executive functions showed *less* impairment in the older group. These results suggest, if anything, a milder impairment in the older than in the younger AwPKU relative to controls, contrary to expectation. However, the following section shows that these results are due to the worse performance of the middle-aged control group rather than to better performance of the PKU group. Table 2S, included in the Supplementary Materials shows the performance of the middle-aged PKU group when considered in terms of normative samples from the literature. The PKU group's performance is close to or better than average in tasks tapping language and memory (WAIS, similarities and vocabulary; Rey words immediate and delayed recall; Animal fluency), but it is lower on the Perseverate errors of the WCST tapping executive functions (O.07 of a SD below average). These contrasts are consistent with previous reported patterns (see the meta-analysis of Romani et al. (2022); [4]).

#### 3.1.2 Age comparison (older vs younger cohorts)

Results for individual tasks comparing the performance of PKU and healthy controls across ages are shown in Supplementary Materials B (Table 3S). Table 3 compares indexes of aging in the domains investigated. They are computed by averaging individual z-scores from the relevant younger group across tasks. Overall, performance is significantly worse in the older groups, both for AwPKU and healthy controls. As expected, speed of processing shows significant decrements across tasks. However, there is no speed-accuracy trade-off, and accuracy in the same tasks also decreases with age. This is true both for PKU and healthy controls. There is no significant decrement with age for executive functions, sustained attention, language, and memory. This is true both for the PKU and the control groups (for no decrement with age in healthy adults in tasks tapping inhibitory control see Erb et al., 2023 [58]). In fact, naming and word spelling tasks tapping crystallized intelligence showed better performance in middle-aged than younger AwPKU consistent with results in healthy aging which see crystallized intelligence continue to improve throughout middle age [52,59,60]. Overall, PKU and healthy controls showed similar effects of aging, but speed of processing showed a marginally larger reduction in the control group than in the PKU group, contrary to expectations.

**Table 3:** Effect of aging on cognitive performance for middle-aged adults with PKU compared to middle-aged healthy controls.

INDEX of aging	Middle-Aged AwPKU vs younger AwPKU			Difference from younger group	Middle-Aged controls vs younger controls			Difference from younger group	Difference age effect PKU vs Controls
	n	Mean z-score	*SD*	p *t*-test	n	Mean z-score	*SD*	p *t*-test	p *t*-test
Speed of processing	19	**−0.71**	*0.81*	**<0.001**	27	**−1.27**	*1.1*	**<0.001**	**0.06**
Accuracy	19	**−0.91**	*1.22*	**<0.001**	27	**−0.98**	*2.3*	**0.03**	0.89
Executive functions	19	−0.13	*0.53*	0.3	27	**−0.23**	*0.8*	0.27	0.75
Sustained attention	18	−0.37	*1.46*	0.27	27	**−0.2**	*1.3*	0.47	0.75
Language	19	−0.11	*0.57*	0.43	27	**−0.2**	*0.6*	0.17	0.62
Memory	19	0.01	*0.37*	0.9	27	**−0.22**	*0.5*	**0.07**	0.12
Overall	**19**	**−0.41**	*0.49*	**<0.001**	27	**−0.58**	*0.6*	**<0.001**	0.34

Indexes aging are calculated by averaging individual participants' z-scores from younger groups. Z scores are calculated as the difference of an individual performance from the corresponding younger referent group (PKU or control) divided by the SD of the referent group.

#### 3.1.3 Effects of age within the middle-aged groups

Age impacts performance negatively only from late adulthood. In our middle-aged groups, age spans several years. It is possible, therefore, that effects of age could be appreciated better within our middle-aged groups. Then, if the effects of age interacts with PKU, this effect may be stronger in the PKU than in the control group.

**Fig. 1.**
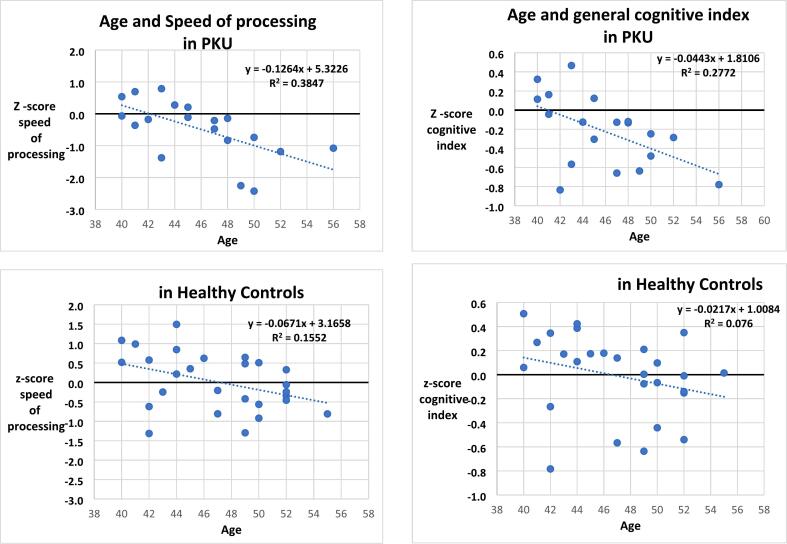
Scatter plots showing the relation between performance (general cognitive index and speed of processing index) and age in the middle-age PKU group and middle-age control group. Z scores from middle-age control group.

We have examined cognitive performance in terms of our general cognitive index and speed of processing index. As expected, in the control group, age correlated significantly with the speed of processing index, with speed decreasing with age (*r* = −39; *p* = .04); instead, age did not correlate with the general cognitive index (*r* = −0.28; *p* = 16). In the PKU group, age correlated strongly with both the speed of processing index (*r* = −62; *p* = .005) and the general cognitive index (*r* = −0.53; *p* = .02). Fig. 1 shows scatter plots of these relationships across groups. Regression lines have a steeper inclination in the PKU group with more units of cognitive performance lost for units of age than in the control group but without differences reaching statistical significance.

To assess statistically whether the detrimental effect of age was stronger in the PKU than in the control group, we used the linear-model option of Jamovi and entered performance as the dependent variable (either the general cognitive index or the speed of processing index), group as a factor and age as a co-variate, and considered the interaction age X group, as well as the main effects. With the general cognitive index, there was a main effect of group because the PKU group performed worse (F = 6.2; *p* = .02; η^2^ = 0.13); a main effect of age because performance decreased with age (F = 8.1; *p* = .007; η^2^ = 0.15) but no significant interaction group x age (F = 0.96; *p* = .33; η^2^ = 0.02).

With the speed of processing index, there was, again, a main effect of group because the PKU group performed worse (F = 6 = 7.8; *p* = .008; η^2^ = 0.16); a main effect of age because performance decreased with age (F = 15.3; *p* < .001; η^2^ = 0.27) but no significant interaction group x age (F = 1.4; *p* = .24; η^2^ = 0.03).

#### 3.1.4 Discussion

The performance of the older PKU group (aged 40+) was significantly and systematically worse than that of a younger PKU group (aged 18–36), showing deterioration with aging. However, the controls showed, if anything, an even stronger effect of aging. It is possible, however, that the performance of the older controls was worse not only because of age but also because of different recruiting and testing conditions which were less strict than those used for the younger controls. The poor performance of the older control group would have reduced any difference with the age matched PKU group. In fact, compared to controls, the middle-aged AwPKU were impaired in speed of processing, sustained attention and overall, but effects were modest and only marginally significant. We will return to this in the general discussion. When we considered the middle-aged groups on their own and assessed the effect of age on performance within each group, we found that, numerically, the association between age and cognitive measures was closer (stronger correlation) and more predictive of change (steeper trend) in the PKU group than in the controls. However, differences did not reach statistical significance and therefore we can only take this trend as suggestive. Our sample is small; testing more participants and combining results with other studies will be important.

### 3.2 Wellbeing

Table 4 compares feeling of depression and anxiety, and effects of the COVID-19 pandemic in our middle-aged AwPKU relative to age-matched controls. AwPKU demonstrated significant more anxiety and depression. Individually, three participants reported anxiety levels above the cut off for moderate anxiety (scores 23, 31, 34), and two of them reported depression levels above the cut off for depression in a non-clinical population (score 24 and 33). Middle-aged AwPKU also reported a more negative impact of lockdown on all mood dimensions compared to controls, with differences reaching significance for anxiety, exhaustion, stress, and worries about their health. People with PKU reported negative effects of the COVID-19 pandemic on all measures in contrast with the controls who indicated a slight improvement in anger and health worries, possibly due to the lockdown reducing the chances of conflict outside the home, and the probability of experiencing other health issues. Finally, middle-aged AwPKU reported that the lockdown created slightly more difficulties in managing PKU and accessing supplements.

**Table 4:** Score of Middle-aged AwPKU and control participants on measures of anxiety, depression, and difficulties during the COVID-19 pandemic.⁎, ⁎⁎

		Middle-Aged AwPKU				Middle-Aged Controls				PKU vs. Controls
	n	Mean	*SD*	Range	z score from controls	n	Mean	SD	Range	*t*-test
**Beck Anxiety Inventory (BAI)**	16	**12.2**	*10.4*	0–34	−1.4	25	**4.8**	5.4	0–22	**0.005**^**⁎⁎**^
**Beck Depression Inventory (BDI)**	16	**10.4**	*8.4*	1–33	−2.0	25	**4.4**	3	0–11	**0.002**^**⁎⁎**^

**COVID-19 questionnaire**										
Anger	16	**0.6**	*1.1*	−2–3	−0.6	25	**−0.1**	*1.2*	−3–2	0.06
Anxiety	16	**1.1**	*1.2*	−1–3	−0.9	25	**0.2**	*1*	−2–2	**0.02**^**⁎**^
Exhaustion	16	**1.1**	*1.1*	−1–3	−0.8	25	**0.0**	*1.4*	−3–3	**0.01**^**⁎⁎**^
Isolation	16	**0.9**	*1*	0–3	−0.3	25	**0.6**	*1.1*	−2–3	0.43
Sadness	16	**0.9**	*1*	−1–3	−0.5	25	**0.3**	*1.1*	−3–2	0.05
Stress	16	**1.3**	*1.2*	0–3	−0.7	25	**0.2**	*1.5*	−3–3	**0.02**^**⁎⁎**^
Worried for health	16	**0.9**	*1.1*	−1–3	−0.9	25	**−0.1**	*1.1*	−3–2	**0.008**^**⁎⁎**^
Overall	16	**0.97**	*1.1*		**−0.68**	25	**0.40**	*1.2*		**0.003**^**⁎⁎**^
**PKU general management**	16	**0.1**	*1.2*	−3–3	–	–	–	–	–	–
**PKU access to supplements**	16	**0.3**	*0.6*	0–2	–	–	–	–	–	–

Higher scores indicate more difficulties/more difficulties during the COVID-19 pandemic. An average score of zero represents no impact of the lockdown.

⁎ *T*-test is significant at the 0.05 level (2-tailed).

⁎⁎ *t*-test is significant at the 0.01 level (2-tailed).

Table 5 compares health-related quality of life using the SF-36 questionnaire in younger and older AwPKU and in younger and older controls. Note however, that only normative results are available for older controls [61] and that results for older PKU participants were collected during the COVID-19 pandemic, thus conflating an effect of aging with that of the pandemic.

**Table 5:** Scores of older and younger PKU participants on the SF-36 health related quality of life questionnaire.

SF-36: (/100)	Middle-aged AwPKU (age: 40–56)				Middle-aged PKU vs controls	Younger AwPKU (age: 18–36)				Younger AwPKU vs controls	PKU Age effect
	n	Mean	*SD*	Range	*t*-test	n	Mean	*SD*	Range	*t*-test	Mean
Physical functioning	15	**87.9**	*14*	60–100	0.09	26	**97.5**	*8*	60–100	0.75	−1.20
Emotional wellbeing	15	**62.3**	*16.4*	40–90	**0.01**	26	**78.9**	*13.7*	47–97	0.23	−1.21
Energy/vitality	15	**41.0**	*15.5*	15–60	**<0.001**	26	**68.1**	*18.2*	38–100	0.09	−1.49
General health	15	**54.3**	*22.3*	20–90	**0.006**	26	**83.8**	*11.1*	56–100	0.04^⁎^ better	−2.66
Health change	15	**46.7**	*16*	25–75	–	26	**70.0**	*19.8*	40–100	0.28	−1.18
Pain	15	**72.8**	*22.5*	35–100	**0.02**	26	**83.6**	*17.3*	73–100	0.16	−0.62
Social functioning	15	**80.8**	*17.6*	62.5–100	0.24	26	**83.4**	*9.8*	90–100	0.33	−0.27
Role limitation/physical functioning	15	**80**	*27.1*	25–100	0.07	26	**95.6**	*13.1*	50–100	0.89	−1.19
Role limitations/emotional functioning	15	**62.2**	*37.5*	0–100	**<0.001**	26	**91**	*16.4*		0.65	−1.76
**AVERAGE**		**66.7**	*22.7*		**0.048**		**84.4**	*6.8*	94.2–71.8	0.08	**−2.60**


	Middle-aged controls Norms (age: 45–54)			Younger controls (age: 18–36)				Controls Age effect
		Mean	*SD*	n	Mean	*SD*	Range	Mean
Physical functioning	297	**87.2**	*20.9*	27	**98.1**	*5.9*	70–100	−1.85
Emotional wellbeing	297	**75.6**	*20.2*	27	**74.5**	*12.8*	43–93	0.09
Energy/vitality	296	**63.0**	*23.1*	27	**60.5**	*13.8*	42–79	0.18
General health	297	**71.3**	*23.4*	27	**76.1**	*14.9*	44–100	−0.32
Health change		–	–	27	**64.4**	*16.9*	20–100	–
Pain	298	**80.7**	*25.5*	27	**89.3**	*11.1*	36–100	−0.77
Social functioning	297	**87.7**	*22.8*	27	**80**	*14.9*	36–91	0.52
Role limitation/Physical functioning	296	**83.5**	*33.8*	27	**97.1**	*10.2*	50–100	−1.33
Role limitations/emotional functioning	297	**88.4**	28.6	27	**88.9**	*18.5*	50–100	−0.03
**AVERAGE**		**79.7**	*24.8*		**80.4**	*9.5*	91.4–58.3	**−0.07**

Lower score = worse outcome.

The middle-aged AwPKU showed significantly worse quality of life compared to norms (*t*-test (322) = 2.9; *p* = .004), likely reflecting the effects of the pandemic. The younger AwPKU showed no difference in quality of life from the controls. Moreover, the middle-aged AwPKU showed significantly worse quality of life than the younger AwPKU reflecting a combined effect of aging and the pandemic. They showed significantly worse scores for all measures except social functioning with a large overall effect size = 2.6. In comparison the combined effects of aging and the pandemic was small in the controls (this was true whether controls tested in the lab were used or normative data effect size =0.25; for the aging effect in controls [62]).

Table 6 shows the results for the PKU QoL questionnaire. Results show on average a modest impact of PKU on quality of life, with scores for all four subdomains ranging from 0.25 to 0.34. However, results show a wide range of scores with some participants reporting a much stronger impact than others. Direct comparisons are difficult but these results appear to show a wider distribution of scores and a stronger impact than that reported for younger sample of AwPKU in other studies (e.g., see Maissen-Abgottspon et al., 2023) [38].

**Table 6:** Results for PKU QoL.

PKU QoL: (/100) Higher score = worse outcome	Middle-aged AwPKU			
	n	Mean	*SD*	Range
Symptoms	16	29.2	*11.8*	14.6–50
PKU general	16	27.4	*10.5*	15.6–53.3
Supplements	14	24.5	*19.1*	0–69.4
Dietary restriction	16	34.1	*21.2*	0–62.4

All subscales normalized to 100. Higher scores were associated with more frequent symptoms, poorer adherence, or a greater impact of PKU on quality of life.

Taken together these results suggest that adults with PKU are more susceptible to a deterioration in quality of life when impacted by negative factors (such as age, or COVID-19).

### 3.3 Relationship to current (most recently recorded) Phe level

We correlated current Phe and Phe in the previous decade of life with our composite cognitive measures and with our wellbeing measures. Correlations were both positive and negative with no systematic pattern. Only one correlation reached significance (less exhaustion during the COVID-19 pandemic was correlated with higher Phe levels). This result would not survive correction of multiple comparisons and it is likely to be a chance result, given the lack of a systematic pattern (see Supplementary Materials B Table 4S). Lack of correlations are not surprising given the small number of participants.

## 4 General discussion

### 4.1 Cognitive outcomes

Our middle-aged PKU group (average age 45 years; SD = 4) was marginally impaired compared to controls with our indexes of speed of processing and sustained attention, but not with accuracy measures, and not with measures of language, memory and executive functions focussing mainly on inhibitory control. These results replicate previous patterns that see AwPKU to be generally slow but accurate, have a problem with sustained attention, not show any impairment in tasks tapping inhibitory control, and do well in language tasks [62]. When all cognitive measures were considered together, our middle-aged PKU group only showed a marginal impairment (z score = − 0.22; *p*=. 05). These results do not show any increased impairment with age when compared to a younger AwPKU group. Size of impairment relative to age-matched control groups was actually numerically smaller in the older group, although not significantly so (younger AwPKU average age 27 years; SD = 9; average impairment z score = −0.40; difference between age groups; *p* = .16).

These results are only partially reassuring. When younger and older PKU cohorts were compared directly (rather than relative to controls), the middle-aged AwPKU performed worse than the younger AwPKU in all domains, with the difference being highly significant in tasks where performance was measured with both RTs and accuracy (speed and accuracy indexes), and overall. It is true that the effect of age –as a difference between middle-aged and younger groups– was, if anything, stronger in the controls than in PKU. However, when we considered the effect of aging within the middle-age groups, thus considering individual differences in age from 40 to 58 years old where effects can be stronger, we found that age correlated more strongly with performance in the PKU group and accounted for more variation, although differences between groups did not reach statistical significance. Taken together these results do not exclude a possible age acceleration in PKU, and point to the challenges posed by cross-sectional comparison between groups.

The lower performance of our middle-age control group compared to the younger group may have been affected not only the difference in age, but also by the less rigorous modalities of recruitment and testing conditions –enforced by online testing– which may have impacted negatively on the older control group, reducing differences with the PKU group. The middle-aged group was tested online while the younger group was tested face-to-face. This has allowed us to recruit the middle-aged controls through social media with the promise of monetary remuneration. These participants may have been less motivated to do well, than the younger control participants who were largely recruited from family, friends, students, and university staff. The online modality does not allow the same strict control on testing conditions. The control participants recruited online, therefore, may have been less careful than the PKU participants to ensure that testing occurred in ideal conditions (away from sources of distraction). This may have negatively affected performance and reduced differences from the clinical group. In fact, the middle-aged controls showed a very large decrement in performance compared to younger controls. In the age range we have tested we would have expected performance to remain more stable [52,59].

In contrast, we may have recruited only middle-aged AwPKU who felt comfortable and technically able enough to cope with online testing. Not very many AwPKU took up our offer to participate in research, representing a small sample of the people treated by either of our participating clinics (considering they were some of the largest clinics in the UK). Thus, it is likely that we have recruited the most motivated, and, potentially, the better performing members of these clinics. This suggests that differences with the younger AwPKU group should be taken seriously and be of potential concern [52,59]. Further studies, carried out in more controlled conditions, with PKU participants recruited more widely, and/or with older cohorts, are needed to reassure us of no accelerated deterioration of cognition in PKU.

### 4.2 Wellbeing

As a group, middle-aged AwPKU showed more difficulties with mental health compared to age-matched controls showing more anxiety, more depression, and exacerbated difficulties dealing with the COVID-19 pandemic. They also showed a sharp reduction in quality of life compared to younger AwPKU. This may be due to increased aging but also to the effects of the COVID-19 pandemic, since the older group was tested during the pandemic. In fact, our findings show a disproportionate impact of the COVID-19 pandemic on the wellbeing of people with PKU. This is contrary to other results showing no increased impact in people with other chronic illnesses [63,64], but consistent with more general results of increased difficulties in the presence of factors increasing stress and vulnerability in the general population and in people with PKU [65,66]. Our results are also consistent with findings from healthy cohorts and PKU cohorts showing reduced wellbeing with increased age. Healthy older adults show progressively more depression and anxiety and reduced quality of life after 50 years of age [62]. Douglas et al. (2013) [48] found that PKU quality of life scores were inversely correlated with age in 37 PKU participants aged 10–49. Bilder et al. (2013) [40] found a positive association between an increase of symptoms and age in 64 AwPKU aged 17–48, using the Brief Symptom Inventory (BSI), with increases in the Global Severity Index, Positive Symptom Distress Index, obsessive-compulsion, depression, and psychoticism sub-domains. Our results are consistent with these results and add to them. Although it is unclear how much to attribute to the COVID-19 pandemic, and how much to increased age, our results clearly indicate reduced wellbeing in middle-aged AwPKU relative to both younger AwPKU and age-matched controls in the face of stressors.

### 4.3 Conclusions

Our results show that a period of social difficulty like that involving the COVID-19 pandemic impacted more the mental wellbeing of middle-aged AwPKU than age-matched healthy participants and that there was a steep decline in quality of life compared to younger AwPKU assessed before the pandemic. We recognize that these results come from a small number of participants and do not allow us to disentangle the impact of aging and the COVID-19 pandemic, but they highlight how people with PKU are an at-risk population. Future studies should quantify how different factors may increase fragility in people with PKU (socio-economic factors, environmental factors, and/or biological factors such as aging) and disentangle their relative contribution.

Regarding cognition, results were mixed. When the two age groups were compared directly, the middle-aged AwPKU performed worse. However, when compared to age-matched controls, the size of impairment was, if anything, numerically smaller in the middle-aged AwPKU; this is because the age effect in the controls was large, with the older cohorts performing much worse. This pattern of results is only partially reassuring. The middle-aged groups were tested online rather than face-to-face. It is unclear why an online modality would have increased the age difference among the PKU participants (if anything the group tested online would have included more confident participants). We could see, instead, how online testing could have increased differences in the controls (if the older participants carried out testing in less ideal conditions), thus reducing estimates of impairment. Moreover, when the effects of age were considered within the middle-age groups, they were numerically stronger in the PKU group than in the controls suggesting a stronger deterioration of cognitive performance with age. Therefore, although our results show no catastrophic deterioration of cognitive performance with aging, more evidence is needed to reassure us that PKU will not interact negatively with aging. Our study tested a small cohort at a still relatively young age. Further studies should build on these results to provide stronger evidence.

From a methodological point of view, our results point to the difficulties of comparing *size* of impairment in cross-sectional studies. Longitudinal studies are, of course, ideal since each participant is a control for themselves, but they are difficult to run because of participant attrition and the obvious difficulties of conducting research over the span of years. Cross-sectional studies, therefore, will continue to have an important role in providing evidence, but our results drive attention to the importance of using groups and conditions which are strictly comparable. Online testing was a necessity during the COVID-19 pandemic, but it has become increasingly popular after that. Most studies have found consistency between cognitive assessments delivered face-to-face and remotely [[67], [68], [69]]. Online testing is practical, and it allows researchers to recruit more widely. Our study, however, points to the need to be extra careful in the recruitment of control participants to make sure that they are responsible and carry out testing in optimal conditions.

## Author statement

Lucie Thomas - Investigation: data collection and analysis; writing the thesis chapter on which the paper is based.

Lynne Aitkenhead - Resources: help with participant recruitment.

Karolina M Stepien - Resources: help with participant recruitment; Writing: reviewing the paper.

Alison Woodall - Resources: help with participant recruitment.

Anita Macdonald - Conceptualization: evolution of ideas: Writing: reviewing the paper.

Cristina Romani - Conceptualization: Ideas; formulation or evolution of overarching research goals and aims; Writing original draft & reviewing the paper; Funding acquisition; Supervision: oversight and leadership responsibility for the research activity planning and execution, main supervisor of Ph.D. student (Lucie Thomas) who carried out the research.

## CRediT authorship contribution statement

**Lucie Thomas:** Writing – original draft, Investigation, Data curation. **Lynne Aitkenhead:** Resources. **Karolina M. Stepien:** Writing – review & editing, Resources. **Alison Woodall:** Resources. **Anita Macdonald:** Writing – review & editing, Conceptualization. **Cristina Romani:** Writing – review & editing, Writing – original draft, Supervision, Methodology, Funding acquisition, Formal analysis, Data curation.

## Declaration of competing interest

None.

## Data Availability

Data will be made available on request.

## Appendix A Supplementary data

Supplementary material Image 1
