# Supplementary material for: Cognition and wellbeing in middle-aged early treated people with phenylketonuria: Preliminary results and methodological lessons
Source: Mol Genet Metab Rep. 2024 Nov 21;41:101160. doi: 10.1016/j.ymgmr.2024.101160 (PMC11617784; doi:10.1016/j.ymgmr.2024.101160)
Supplement: Supplementary file 1 — Supplementary material [file mmc1.docx]

**Supplementary Materials A**

**Assessment Measures**

**Cognitive Measures**

Remote assessments were designed to reflect the face-to-face assessments previously used by Palermo (2017) and Romani (2017; 2019) as closely as possible ^13,14,57^. However, due to the limitations of technologies available, some differences were unavoidable. Tasks used to measure performance in each domain are described below.

IQ

WASI^71^ The vocabulary and similarities subtests from the WASI were administered. Verbal IQ was computed.

Visual attention

*Choice reaction time* – Participants were presented with an arrow pointing to either the left or the right of the computer screen. They were asked to respond by pressing the corresponding arrow on their keyboard as quickly as possible. Reaction time and number of correct responses was scored.

*Detection with distractors* – Participants were asked to respond as quickly as possible when a target (ladybird) was presented on a screen, with or without a distractor (green bug), and not to respond if only distractors (green bugs) were presented. Upon completion of 64 trials, the task was run again but required responses were reversed (i.e., participants were asked to respond for the green bug and not respond for the ladybird). Reaction time and number of correct responses was scored.

*Visual search* – Participants were asked to search for a target (the red ladybird) among several distractors (4, 8, or 12 other bugs) on their screen. They were asked to press the ‘m’ key on their keyboard if the target was present, or the ‘z’ key if it was absent (responses were reversed for left-handed participants). In a feature search condition, distractors were green bugs only. In a conjunction search condition, distractors were both green and red bugs. Reaction time and number of correct responses was scored.

Language

*Picture naming* – Participants were asked to name 120 images, split into 24 semantic categories, presented on the screen, one at a time. They were asked to respond as quickly as possible. Reaction time and number of correct responses was scored.

*Word and non-word spelling* – Participants were given a list of words to spell to dictation. Thirty regular and irregular words were presented, as well as 40 non-words. Number of correct responses was scored.

*Phoneme deletion* – Participants were read a word (e.g. table), and then one sound from within that word (e.g. /t/). They were then asked to repeat back the word without the given sound. The task included 20 trials which resulted in real words (e.g., powder, /d/ = power) and 20 which resulted in non-words (e.g., cabbage, /k/ = abbage). Number of correct responses was scored.

*Spoonerisms* – Participants were read two words. They were asked to exchange the first two sounds and repeat the words back (e.g., bad, sin = sad, bin). The task included 12 trials which resulted in two real words, and 12 trials which resulted in two nonsense words. Number of correct responses was scored.

*Stroop task* – Participants were asked to name the colour of stimuli presented on a screen. In a neutral, condition they were presented with a string of X’s. In a congruent condition, they were presented with a word in the same colour text as the word (e.g., the word ‘Green’ in green text). In an incongruent condition, they were presented with a word in a difference colour text than the word (e.g., the word ‘Blue’ in red text).

Executive function

*WCST* (64-card version^72^) - Participants were asked to correctly match 64 cards with symbols on them with corresponding place-holding cards. Sorting rules (colour, form, or number) were not disclosed to participants, and were changed following 10 correct responses. Number of correct responses was scored.

*Verbal fluency* – Participants were asked to name as many words as possible, either starting with a particular letter or belonging to a specific category, within 1 minute. The task included two letter fluency conditions (with letters ‘c’ and ‘p’) and two semantic fluency conditions (with categories ‘clothing’ and ‘animals’). Number of correct responses was scored.

*Sentence completion task –* In the logical condition*,* participants were asked to complete a sentence with the first word coming to mind (e.g., She didn’t have her watch, so she asked for the *…).* In the illogical condition the sentence had to be completed with a nonsensical word completely unrelated to the sentence. 10 sentences were presented in each condition. Performance was measured both time and errors. Inhibitory control was measured as the difference in performance between the illogical and the logical condition.

*Stroop task* – Inhibitory control was measured through differences in response times and errors between the congruent and incongruent condition (see language tasks).

Sustained attention

*Rapid Visual Information Processing (RVP)* – Participants were asked to detect target sequences of three digits (3-5-7, 2-4-6, or 4-6-8) in a rapidly presented string of digits between ‘1’ and ‘9’. Reaction time and number of correct responses was scored.

Memory and learning

*Digit span* – Participants were asked to repeat back sequences of digits read to them. Lists started at a length of four digits. If the participant recalled more than half of the 10 sequences presented for a given length, they were presented with a longer sequence (up to a maximum of eight digits). Number of correct responses was scored.

*Corsi span* – Participants were presented with nine static, white squares on their screen. A sequence of squares flashed red, immediately after which participants were asked to click on squares in the same order that they flashed. Three trials were presented for each sequence length (from 2 to 9). If the participant responded correctly to at least one trial of a given length, they were presented with the next length, otherwise the task was stopped. Number of correct responses was scored.

*Non-word repetition* – Participants were asked to repeat sequences of non-words. Ten trials were presented for each sequence-length (2, 3, and 4 non-words). The task was stopped if more than half of the sequences for a given length were incorrect. Number of correct responses was scored.

*Rey Auditory Verbal Learning Task* – Participants were read a list of 15 nouns and asked to recall as many words as they could, in any order. The list was repeated five times and participants were asked to repeat what they could remember after each presentation. After a 20-minute delay, participants were asked again to recall as many items as they could remember.

*Verbal paired associate learning* – Participants were presented with nine pictures, each associated with a written non-word. They were then presented with the images again and asked to write down the associated non-word. The task ended when all nine words were recalled correctly, or after five presentations of the whole list. Number of correct responses was scored.

As detailed in Table 2 measures of speed of processing and accuracy were computed by aggregating results in visuo-spatial and language tasks were both were collected.

**Wellbeing Measures**

After completing the cognitive assessment, all participants were asked to complete self-reporting questionnaires assessing depression, anxiety, health-related quality of life, and effects of the COVID-19 pandemic on their health and emotional wellbeing. All questionnaires were delivered through Qualtrics.

*Beck Depression Inventory* BDI-II; ^58^ – Included 21 questions to be answered on a 4-point Likert scale ranging from 0 (symptom absent) to 3 (severe symptoms). The questions probe affective, cognitive, somatic, and vegetative symptoms over the prior 2 weeks, in line with the DSM-IV criteria for major depression. An example of questions is: “*Please read each group of statements carefully, then fill in the circle next to the statement in each group that best describes the way you have been feeling over the past two weeks, including today*”, followed by four possible statements (e.g. “0 – I do not feel sad, 1- I feel sad, 2 – I am sad all the time and I can’t snap out of it, 3 – I am so sad and unhappy that I can’t stand it”). Responses were scored by summing participants’ responses (0-3) to all items, the minimum score being 0 and the maximum being 63. Higher scores reflect more severe symptoms, with scores above 20 indicating depression in non-clinical populations.

*Beck Anxiety Inventory* BAI; ^55^ – included 21 questions to be answered on a 4-point Likert scale ranging from 0 (symptom absent) to 3 (severe symptoms). . The questionnaire assesses anxiety symptoms over the last month. An example of question is the following “*Below is a list of common symptoms of anxiety. Please carefully read each item in the list. Indicate how much you have been bothered by that symptom during the past month, including today, by selecting the button in the corresponding space in the column next to each symptom.*”, followed by a list of symptoms (e.g. “numbness or tingling”) and a Likert scale of possible responses (“Not at all; Mildly, but it didn’t bother me much; Moderately – it wasn’t pleasant at times; Severely – it bothered me a lot”). Responses were scored by summing participants’ responses (0-3) to all items, the minimum score being 0 and the maximum being 63. Higher scores reflect more severe symptoms. A score of 0-21 indicates low anxiety, 22-35 indicates moderation anxiety, and 36+ indicates potentially concerning levels of anxiety.

*36-Item Short Form Health Survey* SF-36; ^54^ – This included a 36 questions to be answered on a 5 point Likert scale and split into eight domains:

1. Physical functioning (e.g., *“During the past 4 weeks, have you had any of the following problems with your work or other regular daily activities as a result of your physical health? Cut down the amount of times you spend on work or other activities”* – Yes, No),

2. Bodily pain (e.g., *“How much bodily pain have you had during the past 4 weeks?”* – None, Very Mild, Mild, Moderate, Severe, Very Severe),

3. Role limitation due to physical health problems (e.g., *“Does your health limit you in these activities? If so, how much? Lifting or carrying groceries”* – Yes, Limited a Lot; Yes, Limited a Little; No, Not Limited at all);

4. Role limitation due to personal or emotional problems (e.g., *“Emotional problems interfered with your normal social activities with family, friends, neighbours, or groups?”* – Not at all, Slightly, Moderately, Severe, Very Severe);

5. Emotional wellbeing (e.g., *“[during the last 4 weeks] Have you been a very nervous person?”* – All of the time, Most of the time, A good bit of the time, Some of the time, A little bit of the time, None of the time),

6. Social functioning (e.g., *“During the past 4 weeks, how much of the time has your physical health or emotional problems interfered with your social activities?”* – All of the time, Most of the time, some of the time, A little bit of the time, None of the time);

7. Energy/fatigue (e.g., *“[during the last 4 weeks] Did you have a lot of energy?”* – All of the time, Most of the time, A good bit of the time, Some of the time, A little bit of the time, None of the time), and

8. General health perceptions (e.g., *“I am as a healthy as anybody I know”* – Definitely true, Mostly true, Don’t know, Mostly false, Definitely false).

Reponses to the questionnaire were scored following the RAND 36-Item Health Survey (version 1.0) guidelines ^73^. Numeric values of responses were recoded so that all responses were scored on a range of 0 to 100 with a higher score representing a more favourable health state. Values for all individual responses were then averaged together to create a single score representing each of the eight domains. Scores were then reversed so that higher scores reflected a worse health state (for accurate comparison with other wellbeing measures).

*PKU Quality of Life Questionnaire* PKU-QoL; ^40^ – This is 65-item questionnaire probing four domains:

1. PKU symptoms (e.g., *“In the past 7 days, I had headaches”* – Never, A little of the time, Sometimes, Often, Very often, *“If you had this, do you think it was related to PKU?”* – Yes, No, I don’t know);

2. PKU in general (e.g., *“In the past 7 days, it was hard to do everything I needed to do for my PKU”* – Never, A little of the time, Sometimes, Most of the time, Always);

3. Administration of Phe-free protein supplements (e.g., *“In the past 7 days, I missed taking some supplements”* – Never, 1 or 2 times, 3 to 5 times, 6 or 7 times, More that 7 times, I don’t take a supplements); and

4. Dietary protein restriction (e.g., *“In the past 7 days, I followed my PKU diet”* – Never, A little of the time, Sometimes, Most of the time, Always, I don’t follow a PKU diet).

Responses to each item of the questionnaire were scored from 0-4. Domain scores were then calculated by summing the response scores and applying a linear transformation to the sum so that all domain scores ranged from 1 to 100. Higher scores were associated with more frequent symptoms, poorer adherence, or a greater impact of PKU on quality of life. Domain scores were only calculated if 70% of the questions in a domain were answered, otherwise the domain score was set as missing. We would take scores ≤ 25 reflect little or no impact, domain scores between 26 and 50 suggest moderate impact, domain scores between 51 and 75 indicate major impact, and domain scores > 75 reflect severe impact (see also Bosch et al., 2015).

*COVID-19 questionnaire* – This questionnaire was created ad hoc to evaluate the effects of the UK-wide lockdown on health management and emotional wellbeing. It included nine questions to be answered on a 7-point Likert scale with 0 indicating no change, scores +1, +2, +3 indicating more difficulties and scores -1,-2, -3 indicating less difficulty. Seven questions probed emotional wellbeing asking about stress, isolation, anxiety, exhaustion, sadness, anger, and concern about their health. Questions were presented in this format: *“Please rate how you feel since the UK-wide lockdown was declared on 23^rd^ March 2020. 1) Stressed” –* Significantly more, Moderately more, Slightly more, No more or less, Slightly less, Moderately less, Significantly less). Overall score 1 to 27 indicated a negative impact of the lockdown on emotional wellbeing, while negative score indicates a positive impact. In addition, there were two further questions regarding management of PKU, and access to supplements during the lockdown. An overall score 1-6 indicating that PKU management had become more difficult more difficult during the lockdown and negative scores -1 to -6 indicating that it had become easier.

**Supplementary Materials B**

**Supplementary Tables**

Table 1S. Clinical comparison. Performance of middle age and PKU relative to age-matched healthy control groups on all cognitive tasks. Z-scores reflect differences from age-matched controls. They have been reversed for some tasks so that lower z-scores always indicate worse performance. The impairment effect is computed by subtracting the mean of the control group from the mean of the controls group and diving by the standard deviation of the control group (Glass’s delta effect size).

AwPKU = Adults with PKU. To aid reading z values below .05 have been highlighted.

* *t*-test is significant at the 0.05 level (2-tailed).

** *t*-test is significant at the 0.01 level (2-tailed).

|  | **Middle-Aged  AwPKU** | | |  | **Middle-Aged  Controls** | | |  | **Effect size: PKU vs Controls** | | | | | |
| --- | --- | --- | --- | --- | --- | --- | --- | --- | --- | --- | --- | --- | --- | --- |
| **SPEED OF PROCESSING** | **n** | **Mean** | ***SD*** |  | **n** | **Mean** | ***SD*** |  | **Middle-Aged PKU** |  | ***t*-test p value** |  | **Younger PKU** | **P value t-test  old vs young** |
| Simple detection RT | 19 | **376** | *70* |  | 27 | **356** | *56* |  | -0.4 |  | .62 |  | -0.3 | .53 |
| Choice reaction RT | 19 | **371** | *52* |  | 27 | **362** | *56* |  | -0.2 |  | .82 |  | -0.7 | **.04*** |
| Detection with distractors RT | 19 | **501** | *87* |  | 26 | **493** | *87* |  | -0.1 |  | .97 |  | -0.6 | .06 |
| Feature search RT | 19 | **699** | *160* |  | 26 | **626** | *109* |  | -0.7 |  | .10 |  | -1.8 | .07 |
| Conjunction search RT | 19 | **1000** | *163* |  | 26 | **879** | *157* |  | **-0.8** |  | **.01*** |  | -1.4 | .12 |
| Stroop RT | 13 | **756** | *83* |  | 15 | **734** | *118* |  | -0.2 |  | .58 |  | -1.2 | **.001**** |
| Continuous Picture Naming RT | 13 | **1006** | *120* |  | 16 | **921** | *185* |  | -0.5 |  | .14 |  | -0.4 | .97 |
| **Average** |  |  |  |  |  |  |  |  | **-0.38** |  | **.05*** |  | **-0.89** | .14 |
|  |  |  |  |  |  |  |  |  |  |  |  |  |  |  |
| **ACCURACY** |  |  |  |  |  |  |  |  |  |  |  |  |  |  |
| Choice RT - % errors | 19 | **2.1** | *2.2* |  | 27 | **1.3** | *2.1* |  | -0.4 |  | .38 |  | -0.1 | .64 |
| Detection with distractors % errors | 19 | **9.8** | *14.0* |  | 27 | **7.8** | *14.4* |  | -0.1 |  | .91 |  | -0.5 | .27 |
| Feature search overall % errors | 19 | **3.1** | *2.9* |  | 26 | **2.0** | *2.6* |  | -0.4 |  | .87 |  | 0.2 | .48 |
| Conjunction search overall % errors | 19 | **3.5** | *3.3* |  | 29 | **5.1** | *3.3* |  | 0.5 |  | .25 |  | 0.2 | .72 |
| Stroop N errors | 15 | **0.18** | *0.3* |  | 24 | **0.24** | *0.6* |  | 0.1 |  | .74 |  | -0.1 | .81 |
| Continuous Picture Naming % errors | 16 | **6.9** | *3.2* |  | 25 | **8.2** | *6.8* |  | 0.2 |  | .41 |  | -0.1 | .56 |
| **Average** |  |  |  |  |  |  |  |  | **-0.02** |  | .97 |  | **-0.28** | .18 |
|  |  |  |  |  |  |  |  |  |  |  |  |  |  |  |
| **EXECUTIVE FUNCTIONS** |  |  |  |  |  |  |  |  |  |  |  |  |  |  |
| WCST Total errors | 19 | **16.4** | *7.6* |  | 31 | **16.5** | *10.6* |  | 0.0 |  | .50 |  | -0.5 | **.04*** |
| Fluency (semantic + phonemic) | 19 | **40.4** | *8.3* |  | 27 | **38.0** | *10.4* |  | 0.2 |  | .41 |  | 0.7 | .41 |
| Illogical- log sentence completion errors | 19 | **17.4** | *18.8* |  | 27 | **19.6** | *22.3* |  | 0.1 |  | .71 |  | - | - |
| Illogical - log sentence completion time | 19 | **1.6** | *1.9* |  | 27 | **1.6** | *1.2* |  | 0.0 |  | .96 |  | - | - |
| Stroop Inc-congruent % errors | 15 | **0.27** | *0.9* |  | 24 | **0.54** | *1.3* |  | 0.2 |  | .34 |  | 0.0 | .39 |
| Stroop incongruent-congruent RT | 12 | **168** | *106.2* |  | 15 | **146** | *96.3* |  | -0.2 |  | .59 |  | -0.3 | .84 |
| **Average** |  |  |  |  |  |  |  |  | **0.06** |  | .37 |  | **-0.04** | .23 |
|  |  |  |  |  |  |  |  |  |  |  |  |  |  |  |
| **SUSTAINED ATTENTION** |  |  |  |  |  |  |  |  |  |  |  |  |  |  |
| RVP RT | 18 | **534** | *249* |  | 25 | **441** | *121* |  | -0.8 |  | .17 |  | -0.5 | .68 |
| RVP %errors | 18 | **17.6** | *17.1* |  | 25 | **11.8** | *14.9* |  | -0.4 |  | .24 |  | -0.8 | .25 |
| **Average** |  |  |  |  |  |  |  |  | **-0.58** |  | **.08** |  | **-0.65** | .77 |
|  |  |  |  |  |  |  |  |  |  |  |  |  |  |  |
| **LANGUAGE** |  |  |  |  |  |  |  |  |  |  |  |  |  |  |
| Vocabulary WAIS subtest | 19 | **67.1** | *8.1* |  | 27 | **65.1** | *7.1* |  | 0.3 |  | 0.37 |  | -0.8 | **<.001** |
| Similarities WAIS subtest | 19 | **39.5** | *4.2* |  | 27 | **38.3** | *5.3* |  | 0.2 |  | 0.44 |  | -0.8 | **.03** |
| Spelling words % errors | 18 | **7.4** | *9* |  | 27 | **3.7** | *6.4* |  | -0.6 |  | 0.11 |  | 0.2 | .11 |
| Spelling nonwords % errors | 19 | **16.2** | *12.2* |  | 27 | **22.4** | *13* |  | 0.5 |  | 0.11 |  | -0.2 | .35 |
| Phoneme deletion % errors | 18 | **8.5** | *8.1* |  | 27 | **11** | *8.8* |  | 0.3 |  | 0.33 |  | -0.4 | **.08** |
| Spoonerism % errors | 18 | **6.3** | *6* |  | 26 | **6.8** | *6.7* |  | 0.1 |  | 0.78 |  | -0.6 | .19 |
| **Average** |  |  |  |  |  |  |  |  | **0.13** |  | **1.0** |  | **-0.42** | .44 |
|  |  |  |  |  |  |  |  |  |  |  |  |  |  |  |
| **MEMORY** |  |  |  |  |  |  |  |  |  |  |  |  |  |  |
| Digit span | 19 | **6.3** | *1.5* |  | 27 | **6.3** | *1.3* |  | 0.0 |  | 0.93 |  | -0.4 | .55 |
| Non-word repetition % errors | 19 | **49.5** | *12.7* |  | 25 | **51.3** | *21* |  | 0.1 |  | 0.74 |  | -0.9 | **.01** |
| Corsi span | 19 | **5.3** | *1.2* |  | 27 | **5.9** | *1* |  | **-0.6** |  | **.05*** |  | 0.3 | .71 |
| Pict non-w paired associates % errors | 18 | **38.5** | *24.3* |  | 27 | **35.4** | *24* |  | -0.1 |  | 0.67 |  | 0.2 |  |
| Pict non-w paired associates delayed recall % errors | 18 | **17.9** | *28* |  | 27 | **23.5** | *23* |  | 0.2 |  | 0.47 |  | 0.4 | .56 |
| Rey A learning % errors | 19 | **25.1** | *12.8* |  | 27 | **21.14** | *11* |  | -0.4 |  | 0.26 |  | -0.5 | .86 |
| Rey A delayed recall % errors | 19 | **22.46** | *17* |  | 27 | **22.22** | *18* |  | 0.0 |  | 0.96 |  | -0.2 |  |
| **Average** |  |  |  |  |  |  |  |  | **-0.13** |  | **.24** |  | **-0.15** | .77 |
|  |  |  |  |  |  |  |  |  |  |  |  |  |  |  |
| **OVERALL** |  |  |  |  |  |  |  |  | **-.22** |  | **.05** |  | **-.40** | <.001 |

Table 2S. Standardized performance of the Middle-ag PKU and control group in a subset of cognitive task.

|  | **Middle-age**  **PKU** | |  | **Middle-aged Controls** | |
| --- | --- | --- | --- | --- | --- |
|  | Mean | *SD* |  | Mean | *SD* |
| Vocabulary T-scores | **59.47** | *8.6* |  | **57.15** | *7.9* |
| Similarities T-scores | **57.00** | *6.05* |  | **55.04** | *8.2* |
|  |  |  |  |  |  |
| Rey 1-5 recall -percentile score | **47.45** | *14.09* |  | **46.89** | *12.2* |
| Rey delayed recall - percentile score | **49.45** | *8.69* |  | **47.47** | *10.9* |
|  |  |  |  |  |  |
| Animal Fluency percentile | **57.11** | *26.16* |  | **49.7** | *33.8* |
|  |  |  |  |  |  |
| WCST total errros - score | **93.63** | *12.13* |  | **95.41** | *14.3* |
| WCST perseverative errros - score | **89.26** | *12.28* |  | **90.3** | *12.9* |

Vocabulary and SimilarityT-scores are from WASI manual (Wechsler, 2011)^71^ and control for age; mean=50; SD=10

Rey percentile scores (mean 50; SD=10) have been derived from Stricker et al. (2021)^74^ which provide a correction for age, sex and education

Animal fluency percentile use the normative data and correction for age and education from Troyer et al. (2000)^75^

Wisconsin Card Sorting Test (WCST) standard scores (mean 100; SD=15) are from relevant manual (Heaton et al., 1993)^72^ and control for both age and education

Table 3S. Age effect. Performance of middle- age vs younger participants. Z-scores reflect differences from the corresponding younger groups (PKU or healthy control). They have been reversed for some tasks so that lower z-scores always indicate worse performance in the older participants. AwPKU = Adults with PKU. Age effect is computed by subtracting the mean of the older group from the mean of the younger group and diving by the standard deviation of the younger group (Glass delta effect size).

|  | **PKU PARTICIPANTS** | | | | | | | | | | | | **HEALTHY CONTROLS** | | | | | | | | | |
| --- | --- | --- | --- | --- | --- | --- | --- | --- | --- | --- | --- | --- | --- | --- | --- | --- | --- | --- | --- | --- | --- | --- |
|  | **Middle-Aged  AwPKU** | | |  | **Young  AwPKU** | | |  | **Age Effect size** | |  | **Middle-Aged  Controls** | | | |  | **Young  Controls** | | |  | **Age Effect size** | |
| **SPEED OF PROCESSING** | **N** | **Mean** | ***SD*** |  | **N** | **Mean** | ***SD*** |  | **z-score** | ***t*-test p value** |  | **N** | | **Mean** | ***SD*** |  | **N** | **Mean** | ***SD*** |  | **z-score** | ***t*-test p value** |
| Simple detection RT | 19 | **376** | *70* |  | 27 | **333** | *55* |  | -0.8 | **.02** |  | 27 | | **356** | *56* |  | 27 | **313** | *57.8* |  | -0.7 | **<.001** |
| Choice reaction RT | 19 | **371** | *52* |  | 33 | **303** | *41* |  | -1.7 | **.04** |  | 27 | | **362** | *56* |  | 27 | **280** | *32.6* |  | -2.5 | **<.001** |
| Detection with distractors RT | 19 | **501** | *87* |  | 27 | **439** | *71* |  | -0.9 | **.02** |  | 26 | | **493** | *87* |  | 27 | **399** | *68* |  | -1.4 | **<.001** |
| Feature search RT | 19 | **699** | *160* |  | 27 | **601** | *173* |  | -0.6 | **<.001** |  | 26 | | **626** | *109* |  | 27 | **402** | *58.9* |  | -3.8 | **.01** |
| Conjunction search RT | 19 | **1000** | *163* |  | 27 | **996** | *224* |  | 0.0 | **.01** |  | 26 | | **879** | *157* |  | 27 | **399** | *68* |  | -7.1 | **.001** |
| Stroop RT | 13 | **756** | *83* |  | 26 | **763** | *113* |  | 0.1 | .84 |  | 15 | | **734** | *118* |  | 27 | **487** | *63.5* |  | -3.9 | **<.001** |
| Continuous Picture Naming RT | 13 | **1006** | *120* |  | 26 | **903** | *135* |  | **-0.8** | **.02** |  | 16 | | **921** | *185* |  | 27 | **842** | *136.5* |  | -0.6 | .25 |
| **Average** |  |  |  |  |  |  |  |  | **-0.66** |  |  |  | |  |  |  |  |  |  |  | **-2.9** |  |
|  |  |  |  |  |  |  |  |  |  |  |  |  | |  |  |  |  |  |  |  |  |  |
| **ACCURACY** |  |  |  |  |  |  |  |  |  |  |  |  | |  |  |  |  |  |  |  |  |  |
| Choice RT - % errors | 19 | **2.1** | *2.2* |  | 33 | **0.5** | *0.8* |  | -2.0 | **<.001** |  | 27 | | **1.3** | *2.1* |  | 27 | **0.4** | *0.8* |  | -1.2 | **<.001** |
| Detection with distractors % errors | 19 | **9.8** | *14.0* |  | 27 | **0.8** | *1.2* |  | -7.5 | **.01** |  | 27 | | **7.8** | *14.4* |  | 27 | **0.55** | *0.6* |  | -12.1 | **.005** |
| Feature search overall % errors | 19 | **3.1** | *2.9* |  | 27 | **1.7** | *2.7* |  | -0.5 | .12 |  | 26 | | **2.0** | *2.6* |  | 27 | **2.3** | *2.3* |  | 0.1 | 0.36 |
| Conjunction search overall % errors | 19 | **3.5** | *3.3* |  | 27 | **2.5** | *2.7* |  | -0.4 | .13 |  | 29 | | **5.1** | *3.3* |  | 27 | **3.3** | *4.6* |  | -0.4 | .24 |
| Stroop N errors | 15 | **0.18** | *0.3* |  | 26 | **0.29** | *0.5* |  | 0.22 | .14 |  | 24 | | **0.24** | *0.6* |  | 27 | **2.3** | *2.3* |  | 0.9 | 0.85 |
| Continuous Picture Naming % errors | 16 | **6.9** | *3.2* |  | 26 | **7.2** | *3.9* |  | 0.1 | .15 |  | 25 | | **8.2** | *6.8* |  | 27 | **3.3** | *4.6* |  | -1.1 | .24 |
| **Average** |  |  |  |  |  |  |  |  | **-1.68** |  |  |  | |  |  |  |  |  |  |  | **-2.3** |  |
|  |  |  |  |  |  |  |  |  |  |  |  |  | |  |  |  |  |  |  |  |  |  |
| **EXECUTIVE FUNCTIONS** |  |  |  |  |  |  |  |  |  |  |  |  | |  |  |  |  |  |  |  |  |  |
| WCST Total errors | 19 | **16.4** | *7.6* |  | 33 | **13.7** | *8.3* |  | -0.3 | .24 |  | 31 | | **16.5** | *10.6* |  | 27 | **11** | *5* |  | -1.1 | **.01** |
| Fluency (semantic + phonemic) | 19 | **40.4** | *8.3* |  | 33 | **39.0** | *11.2* |  | 0.1 | .63 |  | 27 | | **38.0** | *10.4* |  | 27 | **45.9** |  |  |  |  |
| Illogical- log sentence completion errors | 19 | **17.4** | *18.8* |  | - | **-** | *-* |  | *-* | *-* |  | 27 | | **19.6** | *22.3* |  | - | **-** | *-* | *-* | *-* | *-* |
| Illogical - log sentence completion RT | 19 | **1.6** | *1.9* |  | - | **-** | *-* |  | *-* | *-* |  | 27 | | **1.6** | *1.2* |  | - | **-** | *-* | *-* | *-* | *-* |
| Stroop Inc-congruent % errors | 15 | **0.27** | *0.9* |  | 26 | **0.73** | *1.2* |  | 0.4 | .16 |  | 24 | | **0.54** | *1.3* |  | 25 | **0.76** | *1.1* |  | 0.2 | .53 |
| Stroop incongruent-congruent RT | 12 | **168** | *106* |  | 26 | **110** | *78* |  | -0.8 | **.06** |  | 15 | | **146** | *96.3* |  | 25 | **92.7** | *54.3* |  | -1.0 | **.03** |
| **Average** |  |  |  |  |  |  |  |  | **-0.14** |  |  |  | |  |  |  |  |  |  |  | **-0.63** |  |
|  |  |  |  |  |  |  |  |  |  |  |  |  | |  |  |  |  |  |  |  |  |  |
| **SUSTAINED ATTENTION** |  |  |  |  |  |  |  |  |  |  |  |  | |  |  |  |  |  |  |  |  |  |
| RVP RT | 18 | **534** | *249* |  | 33 | **433** | *110* |  | **-0.9** | **.05** |  | 25 | | **441** | *121* |  | 27 | **376** | *110* |  | -0.6 | **.03** |
| RVP %errors | 18 |  | *17.1* |  | 33 | **19.8** | *11.6* |  | 1.7 | **.06** |  | 25 | | **11.8** | *14.9* |  | 27 | **12.8** | *8.7* |  | 0.1 | .76 |
| **Average** |  |  |  |  |  |  |  |  | **0.39** |  |  |  | |  |  |  |  |  |  |  | **-0.2** |  |
|  |  |  |  |  |  |  |  |  |  |  |  |  | |  |  |  |  |  |  |  |  |  |
| **Other LANGUAGE** |  |  |  |  |  |  |  |  |  |  |  |  | |  |  |  |  |  |  |  |  |  |
| Vocabulary WAIS subtest | 19 | **67.1** | *8.1* |  | 33 | **58** | *8.8* |  | 1.0 | **.001** |  | 27 | | **65.1** | *7.1* |  | 27 | **63.3** | *6.9* |  | -0.3 | .36 |
| Similarities WAIS subtest | 19 | **39.5** | *4.2* |  | 33 | **36** | *5.8* |  | 0.6 | **.03** |  | 27 | | **38.3** | *5.3* |  | 27 | **38.9** | *3.6* |  | 0.2 | .65 |
| Spelling words | 18 | **7.4** | *9* |  | 26 | **4.0** | *4.6* |  | -0.74 | .11 |  | 27 | | **3.7** | *6.4* |  | 27 | **5.1** | *5.9* |  | 0.2 | .44 |
| Spelling nonwords | 19 | **16.2** | *12.2* |  | 32 | **13.5** | *8.2* |  | -0.33 | .35 |  | 27 | | **22.4** | *13* |  | 27 | **11.9** | *7.9* |  | -1.3 | **.001** |
| Phoneme deletion % errors | 18 | **8.5** | *8.1* |  | 32 | **14.8** | *13.5* |  | 0.47 | .08 |  | 27 | | **11** | *8.8* |  | 27 | **11.6** | *8.9* |  | 0.1 | .82 |
| Spoonerism % errors | 18 | **6.3** | *6* |  | 27 | **11.3** | *15.4* |  | 0.32 | .19 |  | 26 | | **6.8** | *6.7* |  | 27 | **7.3** | *6.8* |  | 0.1 | .81 |
| **Average** |  |  |  |  |  |  |  |  | **0.23** |  |  |  | |  |  |  |  |  |  |  | **-0.2** |  |
|  |  |  |  |  |  |  |  |  |  |  |  |  | |  |  |  |  |  |  |  |  |  |
| **MEMORY** |  |  |  |  |  |  |  |  |  |  |  |  | |  |  |  |  |  |  |  |  |  |
| Digit span | 19 | **6.3** | *1.5* |  | 33 | **6.1** | *0.8* |  | 0.25 | .55 |  | 27 | | **6.3** | *1.3* |  | 27 | **6.5** | *0.9* |  | -0.2 | .44 |
| Non-word repetition % errors | 19 | **49.5** | *12.7* |  | 27 | **47.8** | *13* |  | -0.13 | .66 |  | 25 | | **51.3** | *21.2* |  | 27 | **39.4** | *9.4* |  | -1.3 | **.01** |
| Corsi span | 19 | **5.3** | *1.2* |  | 33 | **5.4** | *0.8* |  | -0.13 | .71 |  | 27 | | **5.9** | *1* |  | 27 | **5.7** | *0.9* |  | 0.2 | .49 |
| Pict non-word paired associates % errors | 18 | **38.5** | *24.3* |  | 27 | **42.7** | *22.7* |  | 0.19 | .56 |  | 27 | | **35.4** | *23.9* |  | 27 | **46.7** | *24.9* |  | 0.5 | .10 |
| Pict non-w paired associates delayed recall % errors | 18 | **17.9** | *28* |  | 27 | **19.3** | *23.6* |  | 0.06 | .85 |  | 27 | | **23.5** | *22.5* |  | 27 | **29.2** | *24.3* |  | 0.2 | .37 |
| Rey A learning % errors | 19 | **25.1** | *12.8* |  | 27 | **24.1** | *11.4* |  | -0.09 | .78 |  | 27 | | **21.1** | *11.1* |  | 27 | **20.1** | *8.7* |  | -0.1 | .72 |
| Rey A delayed recall % errors | 19 | **22.46** | *17* |  | 27 | **16.5** | *16.7* |  | -0.36 | .60 |  | 27 | | **22.2** | *17.8* |  | 27 | **14.3** | *14.5* |  | -0.5 | .96 |
| **Average** |  |  |  |  |  |  |  |  | **-0.03** |  |  |  | |  |  |  |  |  |  |  | **-0.2** |  |
|  |  |  |  |  |  |  |  |  |  |  |  |  | |  |  |  |  |  |  |  |  |  |
| **OVERALL** |  |  |  |  |  |  |  |  | **-.41** | <.001 |  |  | |  |  |  |  |  |  |  | **-.58** | <.001 |

Table 4S. Peason r correlations of cognitive and wellbeing measures with concurrent and adulthood Phe levels in the middle-aged participants. Current Phe relates to the most recently recorded value.

|  | N | **Current Phe** | *p value* | **Adult Phe** | *p value* |
| --- | --- | --- | --- | --- | --- |
| **Cognitive Indexes** |  |  |  |  |  |
| Speed of processing | 16 | -0.21 | *ns* | -0.22 | *ns* |
| Accuracy | 16 | 0.27 | *ns* | 0.23 | *ns* |
| Executive functions | 16 | -0.12 | *ns* | -0.06 | *ns* |
| **BAI** | 16 | 0.09 | *ns* | 0.09 | *ns* |
| **BDI** | 16 | -0.06 | *ns* | 0.0 | *ns* |
| **COVID-19 questionnaire;**  **higher score = more difficulties during COVID** | | | |  |  |
| Anger | 16 | 0.23 | *ns* | 0.24 | *ns* |
| Anxiety | 16 | -0.12 | *ns* | -0.14 | *ns* |
| Exhaustion | 16 | **-0.45** | 0.08 | **-0.49** | **.05** |
| Isolation | 16 | -0.16 | *ns* | -0.08 | *ns* |
| Sadness | 16 | -0.22 | *ns* | -0.20 | *ns* |
| Stress | 16 | 0.36 | *ns* | 0.26 | *ns* |
| Worried for health | 16 | 0.02 | *ns* | -0.08 | *ns* |
| Overall | 16 | -0.06 | *ns* | -0.1 | *ns* |
| **PKU - general management** | 16 | 0.34 | *ns* | 0.31 | *ns* |
| **PKU - access to supplements** | 16 | 0.08 | *ns* | 0.14 | *ns* |
| **PKU QoL: (/100)**  **Higher score = worse outcome** | |  |  |  |  |
| Dietary restriction | 16 | 0.28 | *ns* | 0.15 | *ns* |
| PKU general | 16 | 0.20 | *ns* | 0.11 | *ns* |
| Supplements | 14 | 0.35 | *ns* | 0.32 | *ns* |
| Symptoms | 16 | -0.09 | *ns* | -0.13 | *ns* |
| **SF-36: (/100)**  **Lower score = worse outcome** | |  |  |  |  |
| Emotional wellbeing | 15 | -0.10 | *ns* | -0.21 | *ns* |
| Energy/fatigue | 15 | 0.33 | *ns* | 0.22 | *ns* |
| General health | 15 | -0.07 | *ns* | -0.1 | *ns* |
| Health change | 15 | 0.32 | *ns* | 0.27 | *ns* |
| Pain | 15 | 0.04 | *ns* | 0.11 | *ns* |
| Social functioning | 15 | -0.24 | *ns* | -0.35 | *ns* |
| Role limitation/Physical functioning | 15 | 0.02 | *ns* | -0.10 | *ns* |
| Role limitations/ Emotional functioning | 15 | -0.07 | *ns* | -0.14 | *ns* |

**References**

70. Weschler D. Wechsler Abbreviated Scale of Intelligence--Second Edition (WASI-II). *APA PsycTests*. 2011;doi:10.1037/t15171-000

71. Heaton RK, Staff P. Wisconsin card sorting test: computer version 2. *Odessa: Psychological Assessment Resources*. 1993;4:1-4.

72. Hays RD, Sherbourne CD, Mazel RMJHe. The rand 36‐item health survey 1.0. 1993;2(3):217-227.

73. Stricker NH, Christianson TJ, Lundt ES, et al. Mayo normative studies: Regression-based normative data for the auditory verbal learning test for ages 30–91 years and the importance of adjusting for sex. *Journal of the International neuropsychological Society*. 2021;27(3):211-226.

74.Troyer AK. Normative data for clustering and switching on verbal fluency tasks. *Journal of clinical and experimental neuropsychology*. 2000;22(3):370-378.
